# The Doctor at Home

**Published:** 1889-02

**Authors:** 


### THE DOCTOR AT HOME.

The Boston Med. and Surg. Jour., under the above head remarks,

A doctor's wife was not long since overheard telling her husband that he was pleasant eveywhere save in his own family; and the doctor admitted that his good nature was so exhausted in his daily visits to his patients that he was irritable when he reached home. Exactly how true the doctor's admission was in that particular instance, it is impossible to say, but it seems as though in the ordinary course of a doctor's existence such a condition might often occur. Physicians certainly meet with many things in their daily rounds that try their tempers. Life is for most of them a constant study how to coax or compel obstinate or ignorant, perhaps silly or even insane, patients to follow the course thought to be for their good. All these troublesome individuals must be reasoned with or influenced by some means to do as they ought. To carry his point the doctor must keep his temper. He usually preserves an outword calm, but if he is naturally quick tempered it is often at the cost of an effort which is exhausting. After such a struggle he reaches home in a state of irritability combined with mental and physical weariness, and under such circumstances it is not easy to meet little home trials with patience.

If he is met with the story that Jack will not mind his mother and that Jane is in disgrace because she did not come straight home from school, and, perhaps, in addition, with a demand that he take some means to discover the cause of a disagreeable odor that has manifested itself somewhere about the house, what wonder if his temper proves inadequate for the moment to meet the strain that comes just at the time when he thought the vexations of the day were over and he might eat his belated and overdone dinner in peace. The family cannot know what the husband and father may have been through during the forenoon. His dearest patient may be slowly sinking, and he is filled with bitter thoughts at the limitations of his art; he has just discovered that a patient whose sense of business honor he thought to be of the very highest has deceived him; or the pathologist has shown his diagnosis to be wrong and all the theories he has built up have come to naught; or, most exhausting of all, he has spent an hour with some half hysterical and wholly sick woman, listening to her complaints and trying to set her right. The ever-recurring annoyances cannot be enumerated. Perhaps the doctor's family needs especial grace to so order the household that the home vexations shall not be allowed to obtrude themselves until after such a time shall allow the strained temper to recover.

Doctors are sometimes reviled by ascetics because they indulge in an occasional cigar after the day's work is over. To what class of beings can the cigar be better adapted than to the doctor? The load of their patient's ills grows light when lifted by the smoke, and their minds recover their wanted buoyancy. Perhaps if the woman whose reproach gave rise to these reflections had waited until the cigar was half smoked before she told of the shortcomings of Jack and Jane, she might have had less occasion to complain of her husband's temper. The patients surely are also benefited by the doctor's cigar; he is able to judge of their ills more philosophically when his over-excited nerves are quieted by the gentle sedative.

